# Accuracy of Actigraphy Compared to Concomitant Ambulatory Polysomnography in Narcolepsy and Other Sleep Disorders

**DOI:** 10.3389/fneur.2021.629709

**Published:** 2021-03-04

**Authors:** Anniina Alakuijala, Tomi Sarkanen, Tomi Jokela, Markku Partinen

**Affiliations:** ^1^Department of Clinical Neurophysiology, HUS Medical Imaging Center, Helsinki University Hospital, University of Helsinki, Helsinki, Finland; ^2^Faculty of Medicine and Health Technology, Tampere University, Tampere, Finland; ^3^Department Neurology and Rehabilitation, Tampere University Hospital, Tampere, Finland; ^4^Department of Neurology, Helsinki Sleep Clinic, Vitalmed Research Center, Helsinki, Finland; ^5^Department of Clinical Neurosciences, Clinicum, University of Helsinki, Helsinki, Finland

**Keywords:** actimetry, sleep quantity, diagnostics, central disorders of hypersomnolence, insufficient sleep

## Abstract

Actigraphy provides longitudinal sleep data over multiple nights. It is a less expensive and less cumbersome method for measuring sleep than polysomnography. Studies assessing accuracy of actigraphy compared to ambulatory polysomnography in different sleep-disordered patients are rare. We aimed to compare the concordance between these methods in clinical setting. We included 290 clinical measurements of 281 sleep laboratory patients (mean age 37.9 years, 182 female). Concomitant ambulatory polysomnography and actigraphy were analyzed to determine the agreement in patients with obstructive sleep apnea, narcolepsy, periodic leg movement disorder, hypersomnia, other rarer sleep disorders, or no organic sleep disorder. Bland-Altman plots showed excellent accuracy, but poor precision in single night results between the two methods in the measurement of sleep time, sleep efficiency, and sleep latency. On average, actigraphy tended to overestimate sleep time by a negligible amount, −0.13 min, 95% confidence interval [−5.9, 5.6] min in the whole sample. Overestimation was largest, −12.8 [−25.1, −0.9] min, in patients with obstructive sleep apnea. By contrast, in patients with narcolepsy, actigraphy tended to underestimate sleep time by 24.3 [12.4, 36.1] min. As for sleep efficiency, actigraphy underestimated it by 0.18 [−0.99, 1.35] % and sleep latency by 11.0 [8.5, 13.6] min compared to polysomnography. We conclude that, in measuring sleep time, actigraphy is reasonably reliable and helpful to be used for a week or two to exclude insufficient sleep in patients with the suspicion of narcolepsy. However, the effectiveness of actigraphy in determining sleep seems to decrease in subjects with low sleep efficiencies.

## Introduction

Polysomnography (PSG) in a sleep laboratory has remained the gold standard in measuring the quality of sleep for decades. Technological advancements have refined the method throughout the years, but also produced alternative methods for sleep measurement that could have many advantages over PSG in terms of price and ease of usability in the habitual sleep environment (1).

One of the advantages of actigraphy (ACG) is that it provides longitudinal sleep data over multiple nights. ACG is less expensive, less cumbersome, and easier to use than PSG. ACG is clearly more accurate in estimating sleep time than sleep logs (2). It is probably the most widespread tool for assessing circadian rhythm sleep-wake disorders (2). In addition, its use in measuring different properties of sleep has increased. In a recent clinical practice guideline, American Academy of Sleep Medicine (AASM) introduced several recommendations about the use of ACG in the assessment of patients suspected with central disorders of hypersomnolence, insufficient sleep syndrome, sleep-disordered breathing, or insomnia disorders (3). These recommendations include the use of ACG integrated with home sleep apnea test devices to estimate total sleep time during recording in patients suspected of sleep apnea, and to monitor total sleep time prior to multiple sleep latency test (MSLT) in patients suspected of narcolepsy (3).

However, compared to PSG, ACG has been reported to be less reliable in recognizing short periods of wake and to overestimate measured sleep times (4). Based on previous studies, the validity of ACG somewhat decreases with the decline of sleep efficiency (2, 5, 6). An essential problem with the field is that ACG algorithms of different manufacturers lack shared technical solutions and terms, precluding direct comparisons (7). Quantitative criteria for the assessment of other aspects of sleep than circadian rhythm by ACG were missing for a long time (8–10).

The recent review by AASM set the clinical significance thresholds for the maximum allowable mean difference and the maximum allowable 95% confidence interval (CI) in sleep time between ACG vs. PSG to 40 min among patients with central disorders of hypersomnolence (2). Of note, the task force identified only one study about the concordance of ACG and PSG prior to MSLT among subjects with the suspicion of narcolepsy or hypersomnia (11), and the threshold was set to the same as in those diseases with more available ACG data, like insomnia and insufficient sleep syndrome. In addition, the threshold in sleep efficiency was set to 5% and in sleep latency to 30 min, but these limits were given only for insomnia patients (2).

Only a limited number of actigraphic studies with small number of subjects has been conducted with patients with central disorders of hypersomnolence. Over a decade ago, a study examined the concordance of ACG and PSG prior to MSLT among subjects with suspicion of narcolepsy or hypersomnia (11). Recently, effects of different sensitivity settings of actigraphy regarding its congruence with PSG among idiopathic hypersomnia patients were studied (12). In young operated patients with craniopharyngioma, in higher risk for narcolepsy, PSG, and ACG were also compared recently (13). Actigraphy in sleep apnea patients has been studied by several groups (8, 14–16).

According to ICSD-3, narcolepsy is divided to narcolepsy type 1 (NT1) or narcolepsy type 2 (NT2). NT1 is caused by a selective destruction of hypothalamic hypocretin-producing neurons, while the etiology of NT2 is unknown and usually not hypocretin-related. Patients with NT2 do not have cataplexy, but the other symptoms—excessive daytime sleepiness, disturbed sleep, and parasomnias—are shared in NT1 and NT2.

We aimed to compare the concordance of ACG and ambulatory PSG in subjects having excessive daytime sleepiness (EDS) or other sleep-related symptoms with or without organic sleep disorders to see if ACG is reliable in all diagnostic groups, with a special interest in narcolepsy.

## Methods

The Helsinki and Uusimaa Ethics Committee approved this study (7/2016). As the study was conducted based on documents completed during normally scheduled patient visits, no written informed consent was required.

### Subjects

Initially, study material consisted of all consecutive concomitant ambulatory PSG and ACG recordings conducted at our sleep laboratory in routine clinical practice in the university hospital during 4.5 years, in total 314 recordings. Some of the PSGs failed and, consequently, we included 290 technically reliable enough sleep studies in the study material. Altogether, the data was gathered from 281 individual subjects with the remaining nine recordings being repeated measurements. Actigraphy recordings did not have any technical problems. Subjects were referred for suspicion of sleep-related breathing or movement disorders, central disorders of hypersomnolence, or parasomnias. The subjects were independent in activities of daily living and did not require the assistance of an aide.

### Measurements

An Actiwatch (Cambridge Neurotechnology Ltd, Cambridgeshire, UK) or a MotionWare (CamNtech Ltd, Cambridge, UK) system were used as the ACG devices in this study. Of note, MotionWare system is the successor to Actiwatch made by the same manufacturer. Thus, all the parameters used in this study are identical, and comparable analysis settings were used. During the preceding afternoon, the subjects were carefully guided and prepared at the sleep laboratory for the ambulatory PSG and concomitant ACG and then sent home to sleep in their own beds for the night. The subjects would then return to the laboratory the next morning to return the equipment. In several cases, especially when a central disorder of hypersomnolence was suspected, the subjects were given the ACG device already a fortnight beforehand to be worn at all times so that their circadian rhythms and sleep time could also be evaluated over multiple nights. If there was no such suspicion, ACG was only studied one night, concomitantly with PSG.

The raw data from the ACG devices was processed using an analysis program provided by the manufacturer. The epoch length was 1 min. The definitions of the used ACG parameters were described in detail previously (17). As in PSG, sleep latency (SL) is the difference between bedtime and sleep start, and sleep efficiency (SE) is the percentage of time spent asleep between bedtime and get up time. Actual sleep time (AST) in ACG and total sleep time (TST) in PSG are analogous, both defined as the amount of sleep between sleep start and sleep end.

An Embla Titanium (Embla, Denver, CO, USA) ambulatory PSG system was used for the PSG measurements. Ambulatory PSG comprised six electroencephalography (EEG) derivations, two electro-oculography channels, submental muscle tonus, airflow by nasal pressure transducer, thoracoabdominal respiratory movements, pulse oximeter, body position, electrocardiogram, and electromyography from tibialis anterior muscles. PSG recordings were set to start about an hour before the subject intended to go to bed and to end well after their estimated get up time in the morning. At home, the subject wrote down the exact time for lights off and lights on and also marked them by pressing a button on the ACG device. Afterwards, the identical analyzed time for both PSG and ACG was the time from lights off to lights on. When central disorders of hypersomnolence were suspected and the subject had the MSLT the following day, they had to get up at least 1.5 h before the start of the test, while most of the subjects were able to sleep as long as they wanted. The PSG data was scored manually by medical specialists with experience in sleep scoring according to international criteria (18, 19).

### Diagnostic Groups

Once the clinical examinations of a subject were complete and a diagnosis was set, the subject was categorized based on the diagnosed organic sleep disorder (if any). Narcolepsy was diagnosed according to ICSD-3 criteria (20). Those patients, who were diagnosed before 2014, were reclassified to NT1 or NT2 according to ICSD-3. Hypersomnia group comprised of idiopathic hypersomnia and other hypersomnia syndromes (ICD-10 code G47.1), and this diagnosis was set strictly according to ICSD-3 criteria (20). Obstructive sleep apnea (OSA) was diagnosed if the apnea-hypopnea index (AHI) had a value higher than 5 per hour. In addition, six patients with narcolepsy also had mild OSA, three had moderate OSA, and one had severe OSA as a co-morbidity, but as it did not affect the results, they were classified to the narcolepsy group. The slight difference in defining AHI according to older and newer hypopnea criteria did not affect the results (18, 19).

Periodic limb movement disorder (PLMD) was diagnosed if the periodic limb movement index during sleep was higher than 15 per hour. Of note, the diagnosis of PLMD cannot be set in the context of any other sleep disorder (20) and our subjects were categorized to this group only if they did not have OSA, narcolepsy, or hypersomnia. To be precise, some subjects in this group had restless legs syndrome (RLS) symptoms while awake together with periodic leg movements while sleeping and their clinical diagnosis was RLS, whereas subjects without RLS symptoms had PLMD (20). We combined these subgroups in the study and focused on PSG findings.

The “Others” group included six patients with NREM parasomnias, three with REM sleep behavior disorder, and two with irregular sleep-wake rhythm disorder. Subjects in the “No sleep disorder” group experienced various degrees of tiredness and/or sleepiness, but no sleep apnea, PLMD, narcolepsy, or hypersomnia were found in the sleep studies. Almost all of them slept objectively too little, and probably also the rest had need for longer sleep than they got. Some had mild depressive symptoms or stress, but no clinical diagnosis of depression or anxiety disorder.

### Statistical Analyses

The statistical analysis of the data was carried out with IBM SPSS® Statistics 24.0 (IBM, Armonk, NY, USA), and Stata/SE 16.1 for Mac (StataCorp, College Station, TX, USA). The normality of variables were tested by inspecting the skewness and kurtosis from histograms and Shapiro-Wilk tests. To evaluate agreement between the two methods, Bland-Altman plots were drawn (21, 22). Due to non-parametric distribution of the majority of mean differences, quintile method and logarithmic transformation as proposed by Bland and Altman were used to determine additional limits of agreement (23, 24). To account for the proportional bias, Bland-Altman plots were adjusted for trend and regression lines as well as Passing-Bablok diagrams are shown (25). Mean differences were also plotted against PSG measures since the PSG is considered a golden standard in these measures. Lin's concordance correlation coefficients (CCC) with 95% CI using z transformation were calculated to investigate association (26).

## Results

The study material comprised actigraphy and PSG recordings done to 281 subjects. Their ages varied between 16 and 90 years (mean 37.9 y) and 62.8% were female. In addition to first-time recordings, PSG was done twice to nine subjects. In six of those cases, the reason was a necessary repetition of an MSLT. In the three remaining cases, the repetition was done to clarify a finding after some minor technical problems. When analyzed separately, the results in the group of repeated recordings were consistent with the other results, and as our aim was to analyze concordance between the methods in different recordings, not subjects, we decided to include those repeated recordings, as well. Altogether, 290 recordings were included in this study. The distribution of diagnosed sleep disorders, which are described in detail in the methods section, is summarized in Table 1.

**Table 1:** Diagnostic groups and subgroups.

**Diagnostic groups and *subgroups***	***N***	**Female**	**Male**	**Mean age (SD), years**
OSA	102	48	54	48.1 (14.2)
Narcolepsy	42	24	18	25.7 (9.1)
*NT1*	*37*	*24*	*13*	*25.7 (9.5)*
*NT2*	*5*	*0*	*5*	*26.1 (5.1)*
PLMD	28	24	4	39.4 (11.9)
Others (parasomnia)	11	4	7	31.8 (12.0)
Hypersomnia	6	4	2	35.3 (19.3)
No organic sleep disorder	101	78	23	33.0 (11.2)
All subjects	290	182	108	37.9 (14.8)

*Number, gender distribution, and age on average (standard deviation) in all diagnostic groups and narcolepsy subgroups (in italics). OSA, obstructive sleep apnea; NT1, narcolepsy type 1; NT2, narcolepsy type 2; PLMD, periodic limb movement disorder*.

**Figure 1:**
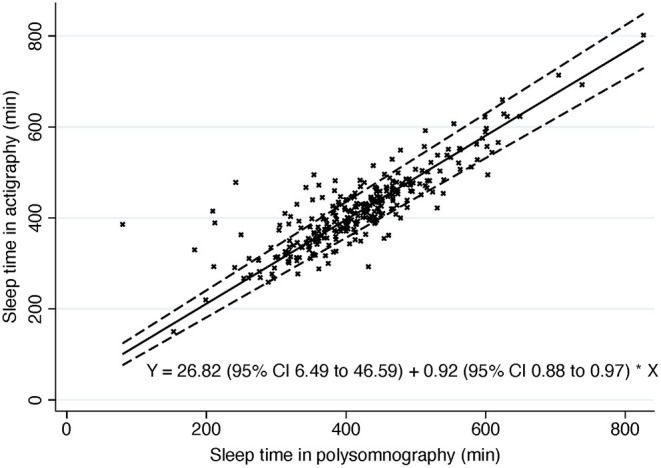
Passing-Bablok of sleep time. Dashed line, 95% confidence interval. Solid line, Passing-Bablok regression line. Y, Passing-Bablok fit.

Mean differences between the ACG and PSG measures followed non-parametric distributions (Shapiro-Wilk *P* < 0.05) in the whole sample and in other subgroups than narcolepsy, NT1, NT2, and hypersomnia. Histograms demonstrated high kurtosis around zero with multiple outliers, which is seen also in Passing-Bablok plot (Figure 1). An extreme outlier e.g., demonstrated a subject with history of Parkinson's disease, restless legs, disturbed nocturnal sleep with only 80 min of total sleep in PSG and 386 min in ACG. Outliers were kept in the sample, and non-parametric methods were used as suggested by Bland and Altman (23). Logarithmic transformation did not change the distribution of mean differences.

### Sleep Time

A priori, we set the clinical significance threshold for mean difference in sleep time to ± 40 min based on the AASM guidelines (2, 3). The mean difference in the whole sample was only −0.13 min (95% CI −5.9 to 5.6) indicating very low bias and high accuracy (Table 2). The mean differences were also in the reference area in all the subgroups. Of all the individual measurements, 70.7% were in this AASM reference area.

**Table 2:** Concordance of sleep time between actigraphy and ambulatory polysomnography.

**Diagnostic groups and *subgroups***	**N**	**Mean difference [95% CI], min**	**Inside AASM limits, %**	**LoA, min**	**CCC [95% CI]**
OSA	102	−12.8 [−25.1, −0.5]	61.8	−135.75, 110.14	0.732 [0.633, 0.808]
Narcolepsy	42	24.3 [12.4, 36.1]	69.0	−50.26, 98.78	0.673 [0.490, 0.800]
*NT1*	*37*	*27.3 [14.1, 40.4]*	*64.9*	*−50.19, 104.73*	*0.655 [0.453, 0.823]*
*NT2*	*5*	*2.0 [−7.4, 11.4]*	*100*	*−12.86, 16.86*	*0.983 [0.849, 0.998]*
PLMD	28	−0.55 [−20.8, 19.7]	71.4	−102.68, 101.57	0.823 [0.658, 912]
Others (parasomnia)	11	23.5 [−7.3, 39.7]	81.8	−23.56, 70.60	0.962 [0.877, 0.988]
Hypersomnia	6	−0.75 [15.4, 16.9]	100	−29.33, 30.83	0.990 [0.933, 0.999]
No organic sleep disorder	101	0.019 [−7.1, 7.1]	77.2	−70.26, 70.30	0.923 [0.889, 0.946]
All subjects	290	−0.13 [−5.9, 5.6]	70.7	−97.49, 97.23	0.845 [0.810, 0.875]

*Mean differences and their 95% confidence intervals in sleep time between ACG and PSG, the proportion of the differences being inside the clinical significant thresholds of AASM, calculatory limits of agreement, and Lin's concordance correlation coefficients and their 95% confidence intervals (P < 0.05 in all) across all diagnostic groups and narcolepsy subgroups (in italics). CI, confidence interval; AASM, American Academy of Sleep Medicine; LoA, limits of agreement; CCC, Lin's concordance correlation coefficient; OSA, obstructive sleep apnea; NT1, narcolepsy type 1; NT2, narcolepsy type 2; PLMD, periodic leg movement disorder*.

**Figure 2:**
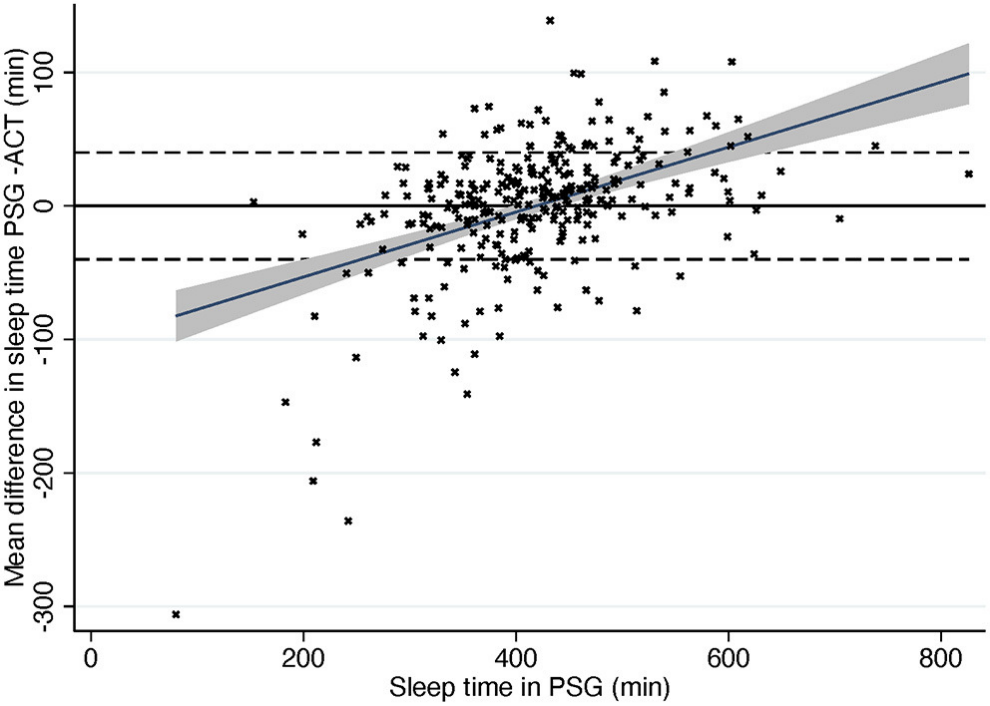
Difference in sleep time compared to total sleep time in PSG. Dashed line, ± 40 min according to AASM criteria. Gray area, 95% confidence interval for regression line. Solid line, mean difference. PSG, polysomnography; ACG, actigraphy.

The Passing-Bablok regression of sleep time showed some but rather low proportional bias in sleep time measures in ACG compared to PSG (slope estimate 0.92; 95% CI 0.88, 0.97) (Figure 1). In other words, there is a trend for actigraphy to overestimate sleep time especially when sleep time is short in PSG with few extreme outliers (Figure 2).

While being very accurate, Bland-Altman plot showed rather wide (−97.49, 97.23) limits of agreement (LoA). Non-parametric distribution and outliers increase limits of agreement that are based on the standard deviation of the bias. In the subgroups where mean differences followed normal distribution (NT1, NT2, narcolepsy, and hypersomnia) LoA were clearly narrower. After removing 5% of the extreme mean differences from both ends—quintile method for non-parametric distributions as suggested by Bland and Altman (23)—LoA in the whole sample were −64.49 (95% CI −70.8, −58.1) and 52.24 (95% CI 46.0, 58.5).

**Figure 3:**
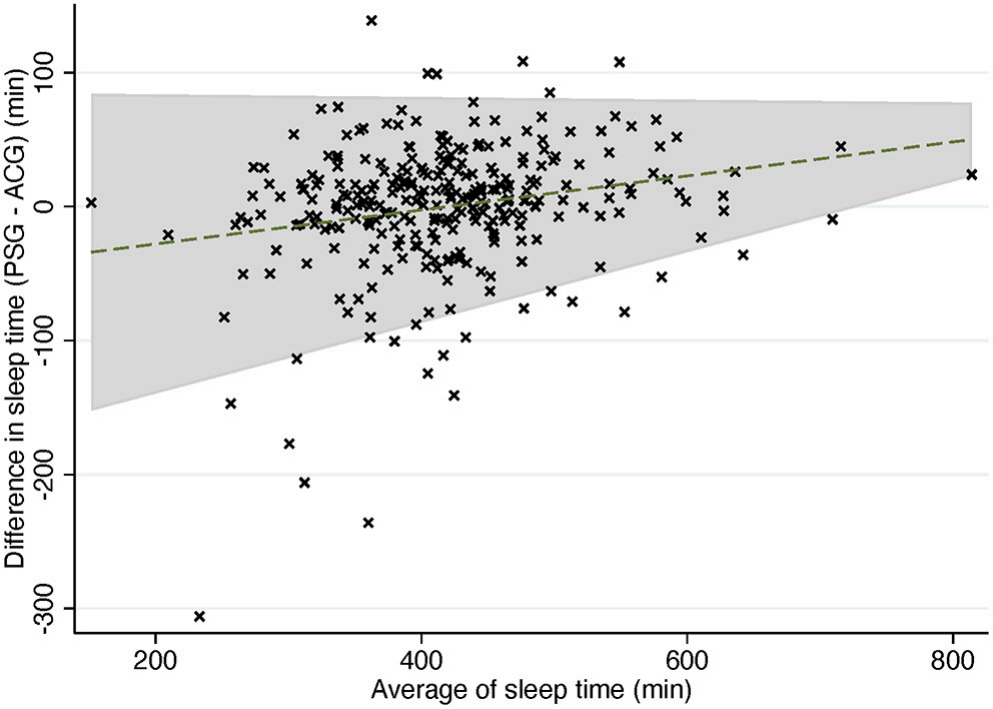
Trend Bland-Altman plot: difference in sleep time compared to average of sleep time in PSG and ACG. Dashed line, mean difference. Gray area, 95% confidence interval for regression line. PSG, polysomnography; ACG, actigraphy.

In trend Bland-Altman plot, the mean difference was −53.26 + 0.13^*^average, and LoA ± 2.46^*^(56.49–0.06^*^average) (Figure 3).

As for concordance correlation, CCC showed high concordance in groups “no organic sleep disorder” and “others (parasomnia)” and also in smaller hypersomnia and NT2 subgroups but poor to moderate in other analyses (Table 2). In total sample, CCC was 0.8454 (95% CI 0.810 to 0.875) which can be considered as moderate.

### Sleep Efficiency

Table 3 depicts the difference of average sleep efficiencies measured by PSG and ACG, i.e., SE in PSG—SE in ACG. Overall, ACG tended to underestimate SE by a very small amount, 0.18, 95% CI [−0.99, 1.35] % in the whole sample. The underestimation was larger, 6.24 [3.42, 9.05] %, in the NT1 subgroup than in the NT2 subgroup. The underestimation was also clear in the others group with mainly patients with parasomnias. By contrast, in patients with OSA, ACG tended to overestimate SE.

**Table 3:** Concordance of sleep efficiency between actigraphy and ambulatory polysomnography.

**Diagnostic groups and *subgroups***	***N***	**Mean difference [95% CI], %**	**Inside AASM limits, %**	**LoA, %**	**CCC [95% CI]**
OSA	102	−2.22 [−4.68, 0.24]	51.0	−26.80, 22.36	0.332 [0.173, 0.475]
Narcolepsy	42	5.63 [3.11, 8.15]	42.9	−10.23, 21.49	0.346 [0.116, 0.540]
*NT1*	*37*	*6.24 [3.42, 9.05]*	*35.1*	−10.31, 22.78	*0.327 [0.085, 0.532]*
*NT2*	*5*	*1.14 [−0.33, 2.61]*	*100*	−1.17, 3.45	*0.931 [0.591,0.990]*
PLMD	28	0.51 [−3.71, 4.72]	53.6	−20.79, 21.81	0.429 [0.124, 0.659]
Others (parasomnia)	11	4.16 [1.89, 6.46]	63.6	−2.57, 10.88	0.644 [0.299, 0.840]
Hypersomnia	6	−0.20 [−3.85, 3.45]	83.3	−7.01, 6.61	0.134 [−0.680, 0.800]†
No organic sleep disorder	101	−0.17 [1.66, 1.32]	63.4	−14.97, 14.62	0.665 [0.547, 0.757]
All subjects	290	0.18 [−0.99, 1.35]	55.5	−19.68, 20.03	0.464 [0.375, 0.544]

*Mean differences and their 95% confidence intervals in sleep efficiency between ACG and PSG, the proportion of the differences being inside the clinical significant thresholds of AASM, calculatory limits of agreement, and Lin's concordance correlation coefficients and their 95% confidence intervals (P < 0.05 in all others except the one marked with †) across all diagnostic groups and narcolepsy subgroups (in italics). CI, confidence interval; AASM, American Academy of Sleep Medicine; LoA, limits of agreement; CCC, Lin's concordance correlation coefficient; OSA, obstructive sleep apnea; NT1, narcolepsy type 1; NT2, narcolepsy type 2; PLMD, periodic leg movement disorder*.

AASM set the clinical the clinical significance thresholds for the maximum allowable 95% CI in sleep efficiency between ACG and PSG to 5% (2). In total, only 55.5% of the measurements were inside these limits. All the CCCs were poor, except in the NT2 group.

### Sleep Latency

As is seen in Table 4, ACG seems to underestimate sleep latency in general, overall on average 11.0, 95% CI [8.5, 13.6] min. The underestimation was the largest, 16.8 [11.1, 22.4] min, in the OSA group. The underestimation was also clear among subjects with no organic sleep disorders. The sole exception to this underestimation by ACG was the narcolepsy group in which the actigraphic SL estimates were very close—on average only 0.049 min longer—to SL in PSG.

**Table 4:** Concordance of sleep latency between actigraphy and ambulatory polysomnography.

**Diagnostic groups and *subgroups***	***N***	**Mean difference [95% CI], min**	**Inside AASM limits, %**	**LoA, min**	**CCC [95% CI]**
OSA	102	16.8 [11.1, 22.4]	84.3	−39,28, 72.82	0.237 [0.121, 0.346]
Narcolepsy	41	−0.049 [−4.1, 4.0]	97.6	−25.34, 25.24	0.352 [0.069, 0.583]
*NT1*	*36*	–*0.2 [*–*4.8, 4.4]*	*97.2*	−26.74, 26.36	0.361 [0.060, 0.603]
*NT2*	*5*	*0.98 [*–*8.5, 10.4]*	*100*	−13.96, 15.92	−0.401 [−0.901, 0.556]†
PLMD	27	8.4 [−1.1, 17.9]	92.6	−38.73, 55.47	0.120 [−0.171, 0.392]
Others (parasomnia)	11	3.2 [−2.6, 9.0]	100	−13.75, 20.20	0.541 [0.039, 0.825]
Hypersomnia	6	3.4 [−4.0, 10.8]	100	−10.38, 17.21	0.835 [−0.474, 0.956]
No organic sleep disorder	100	11.8 [8.7, 14.9]	90.0	−18.97, 42.53	0.787 [0.709, 0.846]
All subjects	287	11.0 [8.5, 13.6]	89.9	−32.22, 54.30	0.520 [0.446, 0.588]

*Mean differences and their 95% confidence intervals in sleep latency between ACG and PSG, the proportion of the differences being inside the clinical significant thresholds of AASM, calculatory limits of agreement, and Lin's concordance correlation coefficients and their 95% confidence intervals (P < 0.05 in all others except the one marked with †) across all diagnostic groups and narcolepsy subgroups (in italics). CI, confidence interval; AASM, American Academy of Sleep Medicine; LoA, limits of agreement; CCC, Lin's concordance correlation coefficient; OSA, obstructive sleep apnea; NT1, narcolepsy type 1; NT2, narcolepsy type 2; PLMD, periodic leg movement disorder*.

AASM set the clinical significance thresholds for the maximum allowable 95% CI in sleep latency between ACG and PSG to 30 min (2). In total, 89.9% of the recordings were inside these limits. All the CCCs were poor.

Men and women as a group did not differ statistically significantly in any of the above-mentioned sleep parameters. Expectedly, older subjects had shorter sleep time, lower sleep efficiency, and longer sleep latency, both in PSG and in ACG. The differences in Bland-Altman analyses or CCC were negligible (data not shown).

## Discussion

For our knowledge, this is the first study comparing ACG and PSG at home. Most people sleep better in familiar environment than in the sleep laboratory (27). As ACG is worn during daily life, including nights at home, this method gives a more exact view of how actigraphy typically performs in different sleep disorders, compared to validation studies with in-lab PSG.

Our results with sleep time were concurrent with previous findings. In previous studies looking at subjects with the suspicion of narcolepsy or hypersomnia, ACG underestimated sleep time by 15.1 min (13) or 15.6 min (11), while our result was 24.3 min for narcolepsy and 0.75 min for hypersomnia group. Further, the slight overestimation of TST by ACG in the OSA group is in line with the recent meta-analysis where ACG was found to overestimate the TST of patients with OSA by 14.5 min (2), while our result was 12.8 min. In our study, accuracy was similar in younger or older subjects, but also opposite results have been shown (28).

The findings in this study suggest that ACG is a reliable tool for estimating TST for many but not all patients with sleep disorders. On average, ACG excelled well in estimating TST with an extremely small bias, only 7.8 s and 95% CI of <6 min. Our mean results fit very well in the clinical significance thresholds for the maximum allowable 95% CI of the mean difference between ACG vs. PSG, set in the recent guidelines by AASM (2). Nevertheless, the large variety of the results is of specific concern. While mean differences between the methods are minor, the results are imprecise in the whole sample. The threshold for maximum allowable 95% CI in the difference in sleep time is too strict for 30% of the measurement pairs in our study. Especially, periodic leg movements and even mild sleep maintenance insomnia seemed to affect the accuracy of sleep time in our subjects.

**Figure 4:**
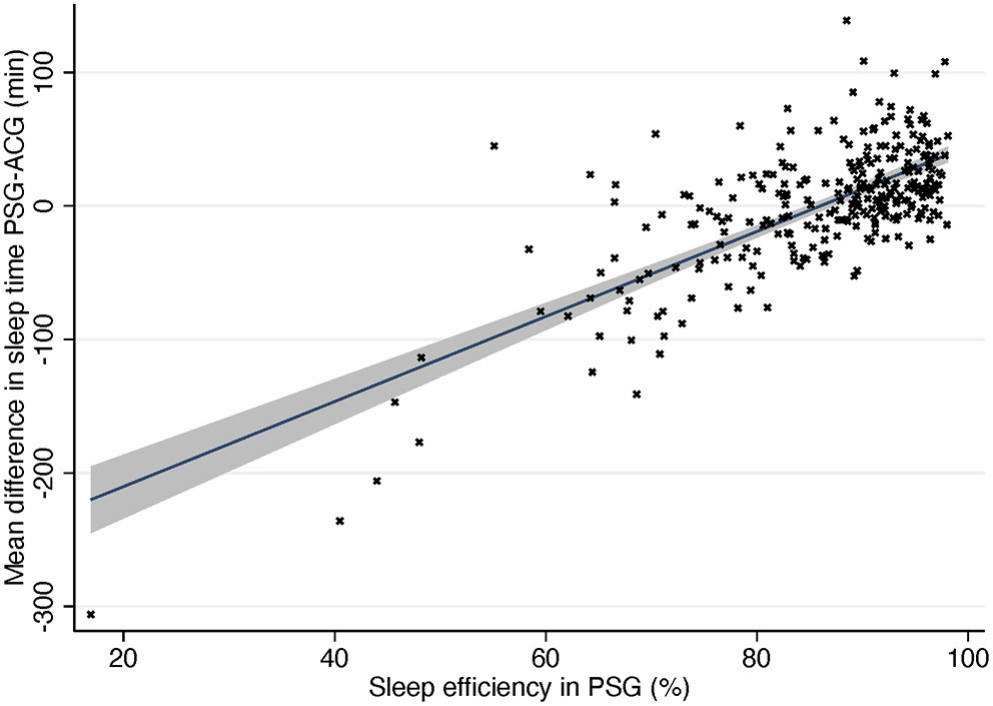
Difference in sleep time compared to sleep efficiency in PSG. Solid line, mean difference. Gray area, 95% confidence interval for regression line. PSG, polysomnography; ACG, actigraphy.

As is seen in Figure 4, actigraphy is accurate when sleep efficiency is high but much less so when sleep efficiency decreases. When a subject has low sleep efficiency, they usually lie immobile but awake, and ACG interprets it as sleep, thus overestimating sleep time heavily. Figure 4 shows also the opposite situation: subjects with normal or good sleep efficiency where ACG underestimated sleep time. Most of these subjects had narcolepsy, especially NT1, or parasomnia. It is a logical finding when the operating principle of actigraphy is taken into consideration. Patients with narcolepsy and/or parasomnia may present abnormal motor behavior in REM and NREM sleep and periodic limb movements (17, 29). ACG would interpret these movements as patients being awake although they would in fact still be asleep.

As for sleep efficiency and sleep latency, there were clear differences between subjects with no sleep disorder and patients having narcolepsy or OSA. Our findings were concurrent with those in previous studies. ACG tends to underestimate sleep latency, probably since subjects usually lie immobile for a while before falling asleep (4). Surprisingly, the narcolepsy subgroup was an exception and was found to have ACG SL measurements that were very concordant with PSG equivalents. This is probably because patients with narcolepsy tend to fall asleep abnormally fast instead of lying immobile but awake. In some cases with narcolepsy, ACG showed even longer sleep latencies than PSG, which may be due to the combination of sleep onset REM and REM without atonia (17, 29). Narcolepsy differs from other sleep disorders in many ways, and the direction of misestimation of sleep time and sleep latency by ACG seems to be one of the differences, at least every now and then.

Periodic leg movements as a disorder or together with another sleep disorder seemed to deteriorate the agreement in all sleep parameters. Since periodic limb movements during sleep are often unrecognized by the patient, it is important to bear in mind this possibility, especially if there are discrepancies in the findings. More research is needed to clarify the effects of sleep-related movement disorders to the reliability of ACG.

To summarize our core findings, ACG shows excellent accuracy i.e., negligible bias or mean difference in sleep time and sleep efficiency, and low bias also in sleep latency. However, the precision of actigraphy in a single night measurement is rather poor as demonstrated by e.g., wide limits of agreement and only moderate number of measurement pairs fitting inside the AASM reference area (24). Still, precision increases with replication and since actigraphy is always done across 7–14 nights, regression toward mean and replication increase precision remarkably.

Insufficient sleep—either behaviorally induced or secondary to some problems with sleep—is a very common cause of EDS and, consequently, the background reason for many patient visits to sleep laboratories. Before conducting more complicated diagnostic tests, such as PSG or MSLT for narcolepsy diagnosis, ACG seems to be a practical tool to show that the cause of EDS could be the lack of adequate amount of sleep related to individual need instead of any specific sleep disorder. ACG is far from being perfect, but there is no better method to assess sleep time across several nights. Actigraphic data is more reliable than data derived from sleep logs (2, 30).

What is more, an actigraphy recording, lasting for 2 weeks or longer, is superior to most other methods in showing delayed sleep phase syndrome (DSPS) or other circadian rhythm sleep-wake disorders. DSPS is common in adolescents and young adults—the same age group where narcolepsy often starts. Actigraphy cannot distinguish between behavioral and genetic DSPS, but it shows the current sleep and wake times. DSPS with very late bedtimes often leads to difficulties in staying awake during the morning hours, and even REM sleep can occur in MSLT sessions if the patient usually sleeps until noon.

To avoid false positive diagnoses of narcolepsy, the use of ACG for at least a week should precede every diagnosis of narcolepsy based on MSLT results (3, 17, 20, 31). This is of the utmost importance for the diagnosis of NT2, where cataplexy and hypocretin deficiency are not found, and the differential diagnosis for insufficient sleep syndrome and DSPS is thus much more difficult than in NT1. Naturally, for a narcolepsy diagnosis, essential symptoms need to be present and all other possible causes excluded (20).

In our clinic, we always use ACG for a fortnight before MSLT so that the last night of ACG recording is the PSG night (ambulatory or in-lab) and we compare the concomitant findings. Consequently, we know if the PSG night was typical for the patient and if the results from the preceding 13 nights of ACG recording were accurate. This procedure substantially increases the reliability of ACG in everyday clinical practice.

Additionally to the help in diagnostics, ACG can be used to assess treatment responses, as the method is known to be highly sensitive in a within-subject design [see Sadeh and Acebo (32)]. There are several illustrative examples of the use of ACG to demonstrate drug-induced changes in PLMD or narcolepsy or CPAP-induced changes in OSA (33–36).

### Limitations

Some limitations of the study need to be acknowledged. Although we had an extensive subject base, which included patients having the most common clinical sleep disorders, we lacked patients with insomnia as their primary diagnosis. Additionally, the number of NT2 and hypersomnia patients were very small, as these diagnoses are rare, when strict diagnostic criteria are used. In comparing concordance of PSG and ACG, we were especially interested in whole-night measures used in clinical practice and we did not do an epoch-by-epoch comparison of the raw data, which would have enabled us to investigate the sensitivity and specificity of ACG in more detail.

### Conclusions

Actigraphy can be a practical tool for measuring some aspects of sleep in situations where PSG is not available for multiple nights or when a full examination is unnecessary to start with. In addition to its established use in circadian rhythm studies, actigraphy is reasonably good at estimating TST. Thus, it is useful in the diagnostic examinations of narcolepsy to see if the subject slept sufficiently during the nights preceding an MSLT for the test to be reliable. However, extreme care should be taken in interpreting the reported values, if the subject has other sleep problems than central disorders of hypersomnolence, as then these values might be misestimated.

## Data Availability Statement

The raw data supporting the conclusions of this article will be made available by the authors, without undue reservation.

## Ethics Statement

The studies involving human participants were reviewed and approved by Helsinki and Uusimaa Ethics Committee. Written informed consent for participation was not required for this study in accordance with the national legislation and the institutional requirements.

## Author Contributions

All authors contributed significantly to the manuscript. AA and TJ contributed to study design, data collection, statistical analyses, interpreting the results, and writing the manuscript. TS and MP contributed to statistical analyses, interpreting the results, and writing the manuscript.

## Conflict of Interest

The authors declare that the research was conducted in the absence of any commercial or financial relationships that could be construed as a potential conflict of interest.

## Abbreviations

AASM: American Academy of Sleep Medicine
ACG: actigraphy
AHI: apnea-hypopnea index
AST: actual sleep time (in actigraphy)
CCC: Lin's concordance correlation coefficient
CI: confidence interval
DSPS: delayed sleep phase syndrome
EDS: excessive daytime sleepiness
EEG: electroencephalography
ICSD-3: International Classification of Sleep Disorders, third edition
LoA: limit of agreement
MSLT: multiple sleep latency test
NT1: narcolepsy type 1
NT2: narcolepsy type 2
OSA: obstructive sleep apnea
PLMD: periodic limb movement disorder
PSG: polysomnography
RLS: restless legs syndrome
SE: sleep efficiency
SL: sleep latency
ST: sleep time
TST: total sleep time (in polysomnography).
